# ADAR-Editing during Ostreid Herpesvirus 1 Infection in *Crassostrea gigas*: Facts and Limitations

**DOI:** 10.1128/msphere.00011-22

**Published:** 2022-04-05

**Authors:** Umberto Rosani, Enrico Bortoletto, Caroline Montagnani, Paola Venier

**Affiliations:** a Department of Biology, University of Padova, Padova, Italy; b IHPE, CNRS, Ifremer, Université Montpellier, Montpellier, France; Stanford University School of Medicine

**Keywords:** ADAR1, hyper-editing, oyster, OsHV-1, antiviral immunity, ADAR, RNA editing, bivalve, innate immunity, malacoherpesvirus

## Abstract

Ostreid herpesvirus-1 (OsHV-1) RNAs are enzymatically modified by A-to-I conversions during the infection of *Crassostrea gigas*. The increase of ADAR1 expression and hyper-editing activity parallel to OsHV-1 RNAs suggests a functional connection between dsRNA editing and antiviral responses. We analyzed 87 RNA-seq data sets from immuno-primed, resistant, and susceptible oysters exposed to OsHV-1 to compare the ADAR hyper-editing levels on host and viral transcripts and trace hyper-editing on the oyster genes. Host RNAs were more hyper-edited than viral RNAs, despite the increased editing of viral RNAs in late infection phases. A set of genes, representing ∼0.5% of the oyster transcriptome and including several tripartite motif-containing sequences, were constantly hyper-edited. Conversely, we identified genes involved in antiviral response, miRNA maturation, and epigenetic regulation that were hyper-edited in specific conditions only. Despite technical and biological bottlenecks that hamper the understanding of the bivalve “RNA editome,” available tools and technologies can be adapted to bivalve mollusks.

**IMPORTANCE** Ostreid herpesvirus-1 (OsHV-1) is a harmful pathogen of bivalve species, such as oysters. However, knowledge is lacking about host–virus interactions at the molecular level, hampering the possibility of a correct management of viral outbreaks and related massive mortalities. Notably, OsHV-1 transcripts are massively modified by host RNA editing enzyme during infection, resulting in multiple A-to-I variations along RNAs assuming double-strand conformations. The impact of these modifications on host transcripts is, however, not completely clear. Analyzing RNA-seq data of oysters infected with OsHV-1, we revealed that ∼0.5% of the oyster transcriptome is always enzymatically modified by ADAR, whereas genes involved in antiviral response, miRNA maturation, and epigenetic regulation were hyper-edited in specific conditions only. Despite our results, relevant technical bottlenecks impair an accurate quantification of RNA editing events, making necessary an approach specifically dedicated to the progressive understanding of oyster “RNA editome.”

## OPINION/HYPOTHESIS

Ostreid herpesvirus-1 (OsHV-1) is pathogenic and even deadly to marine bivalves such as oysters (*Crassostrea gigas*), scallops (*Chlamys farreri*), and clams (*Scapharca broughtonii*) (1). Phylogenetic analyses have classified OsHV-1 and Haliotid herpesvirus-1 (HaHV-1) in a new family (*Malacoherpesviridae*, order Herpesvirales), which includes the only two invertebrate herpesviruses known so far (2). The circulating viral haplotypes likely evolved under the pressure of host immune system (3), mostly involving an interferon-like pathway, autophagy and inhibitors of apoptosis (IAPS) in oysters (4, 5), and IAPS and hemoglobins in blood clams (6). Although mollusks cannot rely on adaptive immunity, transcripts associated with antiviral response and denoting epigenetic functions, like *histone deacetylase 8*, were upregulated until 10 days after immune priming of oysters with polyinosinic:poly(C) (poly(I·C) (7). Moreover, distinct transcriptional profiles characterized the antiviral response of oyster families showing differential susceptibility to OsHV-1, with the resistant oysters displaying a higher basal expression of antiviral genes and a possible quicker transcriptional response (8). Even the viral transcriptome architecture emerges as a possible determinant of host–pathogen interactions, since PacBio RNA data revealed nested gene isoforms, polycistronic genes, and abundant natural antisense transcripts (NATs) in OsHV-1 and HaHV-1, possibly attracting RNA editing activity (9). Adenosine-to-inosine (A-to-I) editing mediated by enzymes of the Adenosine Deaminase Acting on dsRNA (ADAR) family, produce posttranscriptional conversions in RNAs with double-stranded structures (10), a phenomenon relevant in natural and experimental viral infections (11). Although the rules of ADAR1 substrate selection are partially unclear, a massively parallel synthetic approach provided new insights in determining the targets’ editability (12). Mechanistically, dysregulation of ADAR editing in vertebrates has been associated with cancer progression, autoimmune diseases, and increased inflammation, whereas during viral infection the pro- or antiviral role of ADAR hyper-editing depends on the host–virus combination (13). The interferon (IFN)-inducible form of ADAR1 (p150) can block PKR activation, favoring viral replication (e.g., vesicular stomatitis virus), or can impair the replication of viruses by hyper-editing dsRNA structures, leading to the production of nonfunctional proteins among other mechanisms (e.g., measles virus), with dsDNA viruses more rarely impacted by ADAR editing (13). Malacoherpesviruses represent an intriguing exception, since the abundant ADAR activity has probably contributed to viral genome evolution toward a reduction of editable targets (3). However, ADAR’s functional roles in mollusk–malacoherpesvirus combinations remain unclear, and which of the two currently benefits from this editing activity has not been established.

Following a dedicated analysis of RNA-seq data sets of immuno-primed, OsHV-1-susceptible (S) and OsHV-1-resistant (R) oysters infected with OsHV-1, we quantified host and viral ADAR hyper-editing and evaluated the editing impact on the *C. gigas* transcriptome. Then, we discuss the current limitations of this analysis and we trace future research lines, aiming to unravel the “RNA editome” at the edge of host–virus interactions in the marine environment.

## RESULTS

We analyzed 87 *C. gigas* RNA-seq data sets referring to (i) a susceptible oyster family treated with the dsRNA analog poly(I·C) and, therefore, immuno-primed (IP) or with filtered seawater (FSW), and infected with OsHV-1 10 days later (45 samples, EXP1 [7]); (ii) the same susceptible (S) family and a resistant (R) oyster family maintained in cohabitation with infected oysters and monitored for 72 h (hpi; 42 samples, EXP2 [8]) (Table 1, Table S1).

**TABLE 1:** Summary of the analyzed samples and hyper-editing results. Experiment ID, number of samples, sequencing layout, total number of reads and hyper-edited reads, maximal amount of OsHV-1 reads per sample as percentage over total reads, and oyster hyper-editing level are reported. Hyper editing levels have been computed as number of hyper-edited reads over a thousand mapped reads

Expt	No. of sample	Sequencing layout	Total reads (billions)	Total hyper-edited reads	Max. amount of OsHV-1 reads (%)	Oyster hyper-editing level (%)
EXP1	45	2 × 50 bp	3.05	396,375	0.98	0.339 (± 0.036)
EXP2	42	2 × 75 bp	2.36	214,448	0.31	0.173 (± 0.014)

10.1128/msphere.00011-22.1 TABLE S1 Details of the RNA-seq datasets and related analyses. Library ID, description, number of total reads, percentage of reads mapped on the *C. gigas* genome, number of ADAR hyper-edited reads, hyper-editing level in the host, and percentage of reads mapped on the OsHV-1 genome are reported for EXP1 and EXP2 samples. For the samples referring to successful OsHV-1 infections (highlighted in bold), the hyper-editing level in the virus is also reported. Download Table S1, DOCX file, 0.03 MB. Copyright © 2022 Rosani et al. 2022 Rosani et al. https://creativecommons.org/licenses/by/4.0/ This content is distributed under the terms of the Creative Commons Attribution 4.0 International license.

### *C. gigas* ADAR expression correlated with the amount of OsHV-1 RNAs.

**FIG 1:**
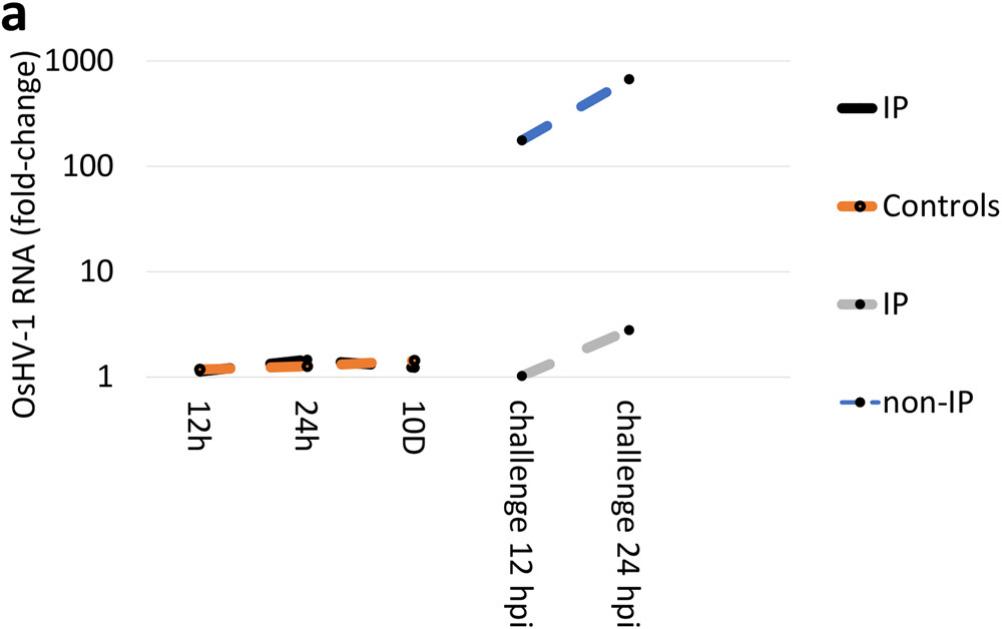
OsHV-1 replication and ADAR1 expression levels. The number of OsHV-1 reads per sample was measured and converted into fold change values (log_10_ scale) in comparison with the paired controls for the EXP1 (a) and EXP2 (b) RNA-seq data sets. The expression levels of ADAR1, measured as transcripts per million (TPM), were similarly converted into fold changes for EXP1 (c) and EXP2 samples (d). For EXP1, the ADAR expression levels of the control and immuno-priming (IP) samples (12h, 24h and 10 days) have been compared to the time zero, whereas during the subsequent OsHV-1 challenge, the IP and non-IP levels have been compared with the paired controls. For EXP2, all the samples have been compared to the time zero samples.

The percentage of reads mapping on the OsHV-1 genome (NCBI ID: KY242785.1) revealed a successful oyster infection, herein defined as samples with at least 0.1% of viral reads, in 6 EXP1 samples and 9 in EXP2 samples (Table S1). In EXP1, the amount of OsHV-1 was 176 and 329 times higher in not immuno-primed (non-IP) oysters compared to the IP oysters, at 12 and 24 hpi, respectively (Figure 1a), with the oysters sampled at 24 hpi showing 0.98% of OsHV-1 reads (mean, *n* = 3; Table S1). In EXP2, both oyster families displayed a bimodal distribution of OsHV-1 reads, which increased 2,268 and 19 times at 24 hpi and then 2,092 and 7.6 times at 60 hpi for S and R oysters, respectively. Susceptible oysters sampled at 60 hpi included 0.31% of OsHV-1 reads (Figure 1b, Table S1). Among the four ADAR genes present in the *C. gigas* genome (NCBI ID: GCF_902806645.1), only ADAR1 (G10242) expression was modulated in EXP1. It increased 14 times at 10 days in IP oysters and reached an expression level of 220 transcripts per million (TPMs; Fig. S1). Both IP and non-IP oysters were subsequently injected with OsHV1 or with FSW. Notably, the high expression level of ADAR1 was maintained at 12 and 24 hpi, with no difference between OsHV-1 injected and paired controls in IP oysters, whereas the ADAR1 expression increased up to 3 times at 24 hpi in the non-IP group related to the paired controls, reaching 47 TPMs (Figure 1c and Fig. S1). In EXP2, ADAR1 expression mirrored the bimodal distribution of OsHV-1 RNAs, with an expression maximum of 173 TPMs at 24 hpi in R oysters and 239 TPMs at 60 hpi in S oysters (Figure 1d and Fig. S2).

10.1128/msphere.00011-22.3 FIG S1 ADAR and ADAR-mediated hyper-editing in EXP1. The expression levels of oyster ADAR1 (dotted line, secondary axis, transcripts per million) together with the level of hyper-editing (histogram, primary axis, % of hyper-edited over mapped reads) are reported for EXP1 samples, averaged per experimental condition (*n* = 3). Download FIG S1, DOCX file, 0.07 MB. Copyright © 2022 Rosani et al. 2022 Rosani et al. https://creativecommons.org/licenses/by/4.0/ This content is distributed under the terms of the Creative Commons Attribution 4.0 International license.

10.1128/msphere.00011-22.4 FIG S2 ADAR and hyper-editing in EXP2. The expression levels of oyster ADAR1 (dotted line, secondary axis, transcripts per million) together with the level of hyper-editing (histogram, primary axis, % of hyper-edited over mapped reads) are reported for EXP2 samples, averaged per experimental condition (*n* = 3). Black and grey refer to samples from susceptible and resistant oysters, respectively. Download FIG S2, DOCX file, 0.05 MB. Copyright © 2022 Rosani et al. 2022 Rosani et al. https://creativecommons.org/licenses/by/4.0/ This content is distributed under the terms of the Creative Commons Attribution 4.0 International license.

### ADAR hyper-editing differentially impacted *C. gigas* depending on experimental conditions.

**FIG 2:**
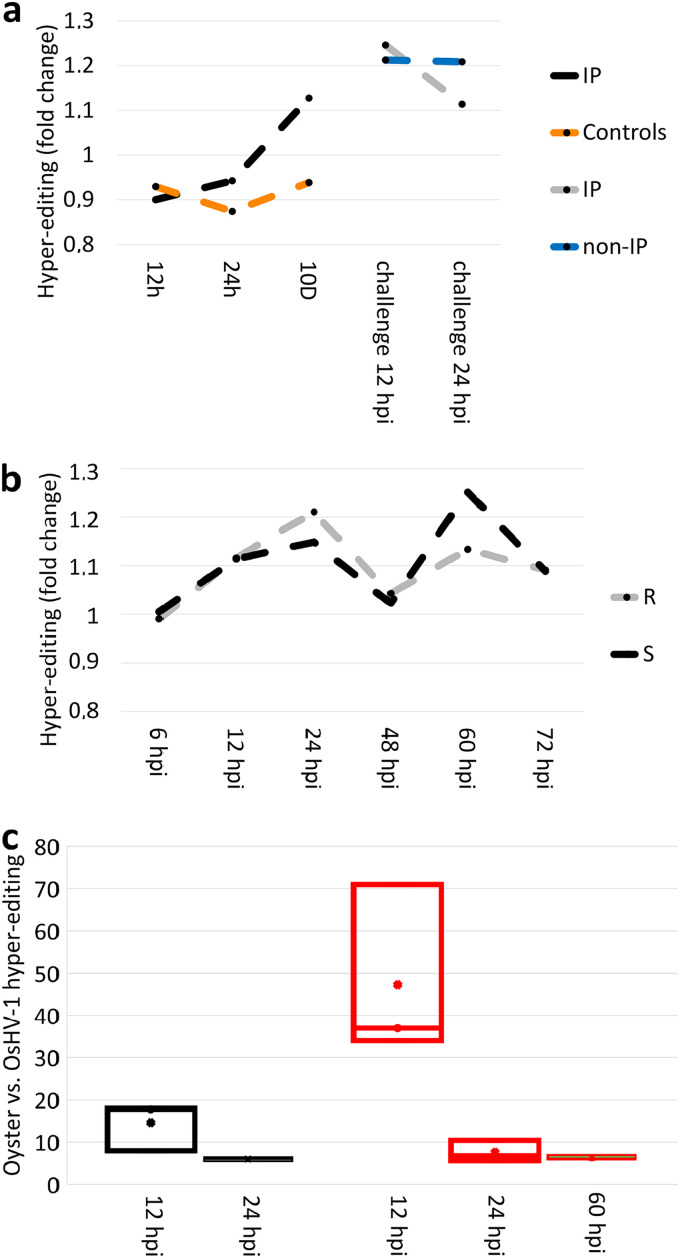
ADAR hyper-editing levels. Fold changes of *C. gigas* hyper-editing values are reported for EXP1 (a) and EXP2 (b) samples. Comparison between *C. gigas-* and OsHV-1-directed hyper-editing is reported for the samples showing productive infections, namely, 12 and 24 hpi for EXP1 (black boxes) and 12, 24, and 60 hpi for EXP2 (red boxes) (c).

We used the *hyperediting* tool (https://github.com/hagitpt/Hyper-editing) (14) to identify *C. gigas* and OsHV-1 hyper-edited reads, namely, reads gathering multiple variations of the same type that, in the case of A-to-G or T-to-C variations, are likely produced by the enzymatic activity of ADAR1. The level of hyper-editing, measured as the percentage of hyper-edited reads over genomic-mapped reads, revealed differences between the two experiments. EXP1 samples displayed a higher hyper-editing level (0.339% ± 0.036) than the EXP2 samples (0.173% ± 0.014), possibly due to the different sequencing layouts (Table 1; Fig. S1 and S2). However, 63% of the hyper-edited reads in both experiments have likely been originated by ADAR1, although a considerable fraction of reads (26–29%) gathering G-to-A variations is detectable (Fig. S3). The hyper-editing levels in EXP1 increased along the immune-priming period, reaching a +20% at 10 days (Figure 2a), followed by a slight increase at 12 hpi of OsHV-1 challenge (+24%), then showed a decrease at 24 hpi (+11%, compared to the initial controls). Non-IP oysters immediately increased hyper-editing levels during OsHV-1 challenge at 12 hpi (+21%) (Figure 2a and Fig. S1). In EXP2, the samples of S and R oyster families showed similar hyper-editing trends, recalling OsHV-1 RNA levels and ADAR1 expression, with an increase up to 25% in S oysters at 60 hpi compared to T0 levels (Fig. S2). S oysters showed lower hyper-editing levels compared to R oysters at 24 hpi (–5%) and higher levels at 60 hpi (+10%, Figure 2b).

10.1128/msphere.00011-22.5 FIG S3 The distribution of the nucleotide variations in hyper-edited reads, for EXP1 (a) and EXP2 (b) samples. Download FIG S3, DOCX file, 0.04 MB. Copyright © 2022 Rosani et al. 2022 Rosani et al. https://creativecommons.org/licenses/by/4.0/ This content is distributed under the terms of the Creative Commons Attribution 4.0 International license.

### Hyper-editing is mostly directed on host RNAs.

In the 15 samples with successful OsHV-1 infection, ADAR-editing impacted more oyster RNAs than OsHV-1 RNAs: 14.6 times at 12 hpi and 6 times at 24 hpi in EXP1; 47 times at 12 hpi, 7.6 times at 24 hpi, and 6.3 times at 60 hpi in EXP2. Notably, ADAR-editing directed on OsHV-1 RNAs increased between 12 and 24 hpi 2.3 times in EXP1 and 6 times in EXP2 (Figure 2c).

### ADAR edited a core set of oyster genes, with some outliers.

**FIG 3:**
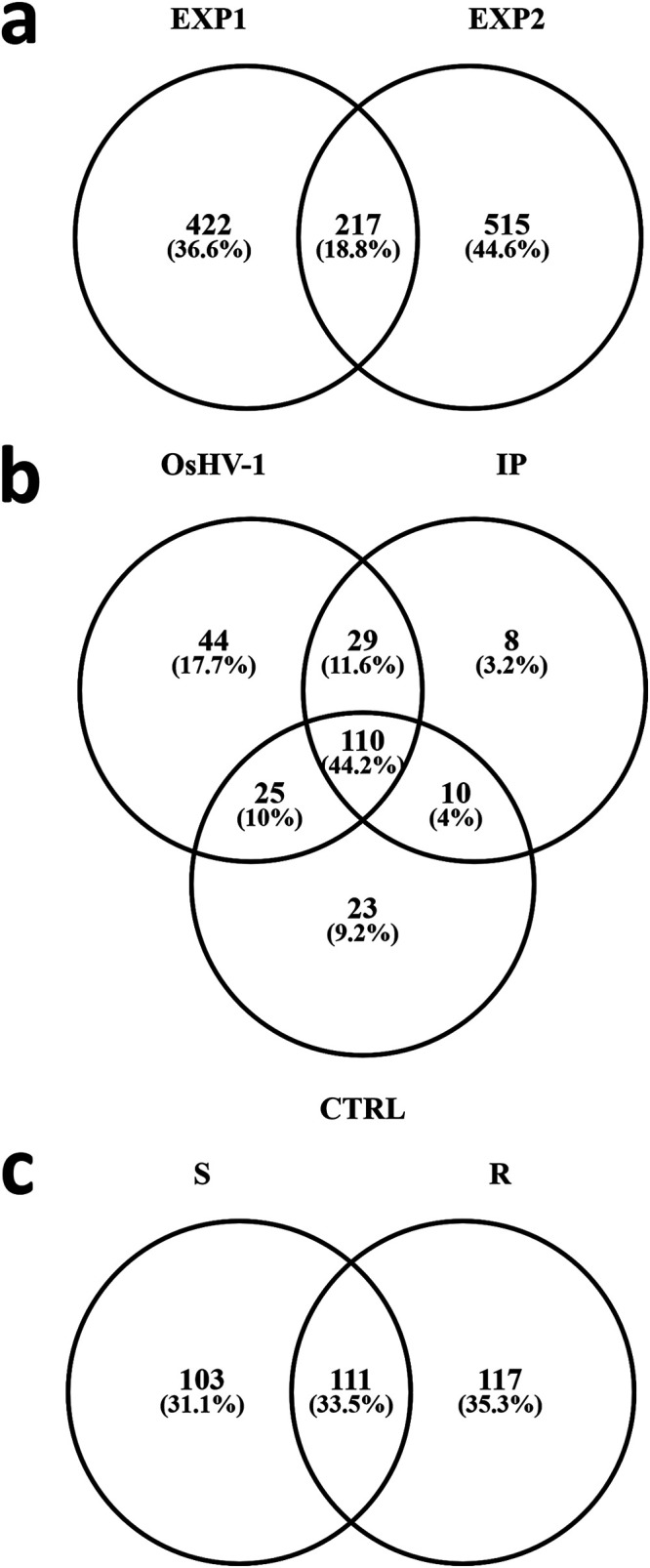
Gene-specific ADAR hyper-editing impacting the oyster transcriptome. The Venn diagram depicts the hyper-edited genes in common or exclusive to EXP1 and EXP2 (a), hyper-edited in all the OsHV-1, IP, or control samples (b), or common or exclusive to S and R oyster families (c).

Setting an arbitrary minimal cutoff of 1 edited read every 1,000 mapped reads to consider a gene “hyper-edited,” we detected 100–206 (total 639) and 158–241 (total 732) *C. gigas* genes consistently edited in the three biological replicates per condition, in EXP1 and EXP2, respectively. Intriguingly, 217 genes (18.8%, Figure 3a) were hyper-edited in both experiments, including 11 tripartite motif-containing proteins (TRIMs), heat shock protein 70 (HSP70), interferon induced protein-44 (Ifi44) and double-stranded RNA-specific editase-1 (ADARB1) among others. In EXP1, we detected 208, 157, and 168 oyster genes hyper-edited in all the OsHV-1, IP, or control samples, respectively, with most of these genes being hyper-edited in all the samples, probably representing physiological hyper-editing targets (44.2%; Figure 3b). Some genes commonly edited in OsHV-1 and IP groups pertained to the antiviral responsive pathways, like ISGs (Ifi44), components of the IL-17 pathway (CIKS), and the dsRNA receptor ZNFX-1. Intriguingly, the ISG *sacsin* was hyper-edited in OsHV-1 and uninfected groups, but not in the IP group (Table S2). In EXP2, we observed an almost equal distribution of genes constantly hyper-edited in S, R samples, or in all samples (Figure 3c). Among R-exclusive genes, we found ADARB1, an IAP, genes involved in miRNA maturation pathway (e.g., lin-41 and RdRP), caspase-7, and cathepsin Z, among others (Table S2).

10.1128/msphere.00011-22.2 TABLE S2 Hyper-editing impact on *C. gigas* genes. The table lists hyper-edited genes in EXP1 and EXP2 with gene ID, description, hyper-editing status in EXP1 and EXP2, and the corresponding normalized hyper-editing levels as reported averages for experimental conditions. [file .xlsx]. Download Table S2, XLSX file, 0.2 MB. Copyright © 2022 Rosani et al. 2022 Rosani et al. https://creativecommons.org/licenses/by/4.0/ This content is distributed under the terms of the Creative Commons Attribution 4.0 International license.

## DISCUSSION

By tracing ADAR hyper-editing along OsHV-1 infection in *C. gigas*, we demonstrated that in the early stage of infection host RNAs are more impacted than viral RNAs, with viral-directed editing increasing along the time course and suggesting that at later stages ADAR1 will mostly target viral dsRNAs. This increase is in agreement with data reported demonstrated for OsHV-1 infection in *S. broughtonii* (9), and likely mirrors the different abundances of viral and host dsRNAs during infection, with the latter increasing as a consequence of sense–antisense gene pair cotranscription (9). Next, we showed that host hyper-editing levels varied along each experiment. Arguably, ADAR activity depends upon several factors: dsRNA availability, amount of functional protein, protein–protein interactions, and chemico-physical cell conditions. In the context of a viral infection, the amount of dsRNA is viral-dependent rather than host-dependent and cellular chemical-physical conditions might change to promote inflammation and/or apoptosis. The sustained expression of oyster ADAR1 up to 10 days after IP suggested a continuous protein production, as a possible counteraction of the instability of ADAR, as showed for human protein due to SUMOylation (15). The absence of ADAR1 in proteomic data of OsHV-1 infected oysters (16) possibly confirmed a rapid degradation. Although protein–protein interactions are mostly unknown in bivalves, it is possible that proteins other than ADAR1 can either interact with it or compete for the same dsRNA substrates, as shown for the enzymatic inactive human ADARB2 (13). Despite these variables, IP oysters maintained stable hyper-editing levels along OsHV-1 infection, whereas R oysters showed higher hyper-editing at the first infection peak compared to S oysters, and lower levels at the second peak. This likely indicates that OsHV-1 resistant and immuno-primed oysters are trained/prone for hyper-editing, even at early stages of infection. Differently, non-IP oysters rapidly reached the same hyper-editing levels of IP oysters at 12 hpi, suggesting that OsHV-1 is highly effective in stimulating hyper-editing, probably because of its peculiar transcriptional architectures (9). A considerable fraction of the oyster transcriptome (∼0.5%) was hyper-edited irrespective of the experiment or condition. The hyper-editing of several TRIMs possibly depends on their ability to interact with the RNA recognition motif of ADAR(s) (17), while still possibly exerting antiviral recognition functions (18). The consistent hyper-editing of some antiviral genes in IP and OsHV-1-treated groups suggests functional interactions, although the biological significance remains unclear and awaits dedicated studies. Differential hyper-editing between resistant and susceptible oysters further supported the functional connection between ADAR hyper-editing and genome-encoded antiviral resistance, both in the timing of ADAR activity, early in infection, and in the hyper-edited substrates, including elements of key transcriptional regulator pathways (e.g., miRNA or epigenetic regulation). Overall, hyper-editing results are consistent with transcriptional observations on resistant versus susceptible and primed versus non-primed oysters: based on the same molecular tools, these oysters can mount antiviral responses with different effectiveness.

### Limitations of the analysis of the bivalve “ADAR editome.”

Several bottlenecks currently limited the analysis of ADAR editing from bivalve RNA-seq data. Firstly, we rely only on a reference genome for the quantification of ADAR editing, not covering interindividual variability, which is extremely important in bivalves, compared to vertebrates, and involves presence–absence of hundreds to thousands of genes (19). Second, the different strategies adopted for the RNA library preparation likely biased the identification of ADAR edits, with the polyA depletion showing a lower efficiency than the ribosomal rRNA depletion in collecting key substrates such as ncRNAs (20). Lastly, available data sets designed for purposes other than the “RNA editome” are suitable for a preliminary evaluation only, due to the lack of paired DNA-seq data, relatively limited number of samples, and a general heterogenicity.

### How future research can improve bivalve RNA editomics.

Several relevant questions regarding computational and biological aspects remain to be answered and challenge future research toward a deeper understanding of the bivalve ADAR editome. How precise is the quantification of ADAR edits and what is the penetrance of these variations? Are other RNA editors active in oysters and can their activity explain the abundance of G-to-A variations (Fig. S3)? What is the fate of the edited transcripts and how do these regulate host/virus interactions? And how is ADAR1 interconnected within the oyster proteome? We highlight the necessity of dedicated projects, which could leverage on existing approaches and implement them to species having specific genome structure, plasticity, repeat contents, and distributions, as bivalves (21). By producing individual paired DNA/RNA-seq data, useful to discriminate ADAR edits from genomic/transcriptomic variations, it will be possible to develop dedicated SNP databases, such as the human/mouse REDI portal (22). The use of rRNA-depleted, stranded RNA libraries can allow the identification of strand-specific edits beyond coding genes and improve the identification of ADAR-editing versus other variation sources. With this regard, CRISPR-based strategies for rRNA depletion (23) could provide the flexibility required for nonmodel organisms. Moreover, sequencing of native RNAs with nanopore (24) could outperform the chemical erasing approach to validate ADAR edits. Finally, the production of recombinant proteins and the development of functional assays could verify ADAR performances and roles in physiology and disease, greatly promoting its functional understanding. Likewise, the integration of promising SNP array results (25) with RNA SNPs can improve the definition of relevant QTLs for disease resistance and other aquaculture-relevant traits.

In conclusion, available tools are ready to be adapted to bivalve mollusks for a deeper understanding of the significance of ADAR activity.
