# Supplementary material for: ADAR-Editing during Ostreid Herpesvirus 1 Infection in Crassostrea gigas: Facts and Limitations
Source: mSphere. 2022 Apr 5;7(2):e00011-22. doi: 10.1128/msphere.00011-22 (PMC9044936; doi:10.1128/msphere.00011-22)
Supplement: TABLE S1 [file msphere.00011-22-st001.docx]

**Supplementary Table 1.** Details of the RNA-seq datasets and related analyses. Library ID, description, number of total reads, percentage of reads mapped on the *C. gigas* genome, number of ADAR hyper-edited reads and hyper-editing level in the host, percentage of reads mapped on the OsHV-1 genome are reported for EXP1 and EXP2 samples. For the samples referring to productive OsHV-1 infections (highlighted in bold), the hyper-editing level in the virus are also reported.

| **Library ID** | **Description** | **total reads** | **% mapped on *C. gigas*** | **ADAR hyper-edited** | **Hyper-editing level (host)** | **% mapped on OsHV-1** | **Hyper-editing level (virus)** |
| --- | --- | --- | --- | --- | --- | --- | --- |
| **EXP1** |  |  |  |  |  |  |  |
| 4_S4_L002 | T0_1 | 4,5E+07 | 68,3 | 10385 | 0,337 | 0,001 |  |
| 5_S5_L002 | T0_2 | 3,6E+07 | 68,8 | 8341 | 0,336 | 0,001 |  |
| 6_S6_L002 | T0_3 | 3,1E+07 | 69,0 | 7045 | 0,331 | 0,001 |  |
| 8_S7_L002 | 12h_post_priming_Poly_I:C_1 | 3,2E+07 | 68,6 | 6437 | 0,297 | 0,001 |  |
| 9_S8_L002 | 12h_post_priming_Poly_I:C_2 | 3,9E+07 | 68,2 | 7640 | 0,290 | 0,001 |  |
| 11_S9_L002 | 12h_post_priming_Poly_I:C_3 | 5,2E+07 | 66,7 | 10970 | 0,317 | 0,001 |  |
| 12_S10_L003 | 12h_post_priming_FSW_1 | 3,2E+07 | 68,1 | 6744 | 0,311 | 0,001 |  |
| 13_S11_L003 | 12h_post_priming_FSW_2 | 2,8E+07 | 67,7 | 5937 | 0,309 | 0,001 |  |
| 15_S12_L003 | 12h_post_priming_FSW_3 | 3E+07 | 67,8 | 6434 | 0,314 | 0,001 |  |
| 23_S13_L003 | 24h_post_priming_Poly_I:C_1 | 3,2E+07 | 66,4 | 6582 | 0,312 | 0,001 |  |
| 24_S14_L003 | 24h_post_priming_Poly_I:C_2 | 4,7E+07 | 67,5 | 9959 | 0,312 | 0,001 |  |
| 25_S15_L003 | 24h_post_priming_Poly_I:C_3 | 3,3E+07 | 67,5 | 7130 | 0,323 | 0,001 |  |
| 26_S16_L004 | 24h_post_priming_FSW_1 | 3,1E+07 | 66,5 | 5754 | 0,278 | 0,001 |  |
| 27_S17_L004 | 24h_post_priming_FSW_2 | 2,8E+07 | 68,0 | 5393 | 0,286 | 0,001 |  |
| 28_S18_L004 | 24h_post_priming_FSW_3 | 5,1E+07 | 67,9 | 10823 | 0,314 | 0,001 |  |
| 60_S19_L004 | 10J_post_priming_Poly_I:C_1 | 2,7E+07 | 68,8 | 7047 | 0,374 | 0,001 |  |
| 61_S20_L004 | 10J_post_priming_Poly_I:C_2 | 4,6E+07 | 68,3 | 12135 | 0,384 | 0,001 |  |
| 62_S21_L004 | 10J_post_priming_Poly_I:C_3 | 2,7E+07 | 68,6 | 6861 | 0,374 | 0,001 |  |
| 64_S22_L005 | 10J_post_priming_FSW_1 | 4E+07 | 67,9 | 8258 | 0,308 | 0,001 |  |
| 65_S23_L005 | 10J_post_priming_FSW_2 | 3,4E+07 | 67,3 | 7105 | 0,315 | 0,001 |  |
| 66_S24_L005 | 10J_post_priming_FSW_3 | 4E+07 | 66,4 | 8471 | 0,321 | 0,001 |  |
| 74_S25_L005 | 12h_post_challenge_Poly_I:C_+_OsHV-1_1 | 3E+07 | 68,0 | 7805 | 0,377 | 0,001 |  |
| 75_S26_L005 | 12h_post_challenge_Poly_I:C_+_OsHV-1_2 | 3,8E+07 | 67,2 | 9339 | 0,362 | 0,001 |  |
| 76_S27_L005 | 12h_post_challenge_Poly_I:C_+_OsHV-1_3 | 2,4E+07 | 68,1 | 6397 | 0,392 | 0,001 |  |
| 78_S28_L006 | 24h_post_challenge_Poly_I:C_+_control_1 | 4E+07 | 67,9 | 10574 | 0,385 | 0,001 |  |
| 79_S29_L006 | 24h_post_challenge_Poly_I:C_+_control_2 | 3,4E+07 | 68,6 | 9142 | 0,391 | 0,001 |  |
| 80_S30_L006 | 24h_post_challenge_Poly_I:C_+_control_3 | 4,3E+07 | 68,6 | 11044 | 0,377 | 0,001 |  |
| **85_S31_L006** | **12h_post_challenge_FSW+_OsHV-1_1** | **3,5E+07** | **68,6** | **7967** | **0,336** | **0,148** | **0,019** |
| **86_S32_L006** | **12h_post_challenge_FSW+_OsHV-1_2** | **3,3E+07** | **72,1** | **6359** | **0,266** | **0,274** | **0,015** |
| **87_S33_L006** | **12h_post_challenge_FSW+_OsHV-1_3** | **2,7E+07** | **66,8** | **5415** | **0,305** | **0,170** | **0,038** |
| 89_S34_L007 | 12h_post_challenge_FSW+_control_1 | 4,1E+07 | 67,8 | 8979 | 0,323 | 0,001 |  |
| 90_S35_L007 | 12h_post_challenge_FSW+_control_2 | 2,6E+07 | 68,5 | 5483 | 0,312 | 0,001 |  |
| 91_S36_L007 | 12h_post_challenge_FSW+_control_3 | 2,9E+07 | 68,0 | 6254 | 0,317 | 0,001 |  |
| 102_S37_L007 | 24h_post_challenge_Poly_I:C_+_OsHV-1_1 | 2,5E+07 | 68,3 | 5937 | 0,351 | 0,001 |  |
| 103_S38_L007 | 24h_post_challenge_Poly_I:C_+_OsHV-1_2 | 3,6E+07 | 68,1 | 9405 | 0,384 | 0,008 |  |
| 104_S39_L007 | 24h_post_challenge_Poly_I:C_+_OsHV-1_3 | 2,8E+07 | 68,4 | 7291 | 0,384 | 0,001 |  |
| 106_S40_L008 | 12h_post_challenge_Poly_I:C_+_control_1 | 2,8E+07 | 64,1 | 7259 | 0,399 | 0,001 |  |
| 107_S41_L008 | 12h_post_challenge_Poly_I:C_+_control_2 | 2,7E+07 | 67,0 | 6378 | 0,346 | 0,001 |  |
| 108_S42_L008 | 12h_post_challenge_Poly_I:C_+_control_3 | 2,9E+07 | 68,3 | 8044 | 0,403 | 0,001 |  |
| **114_S20_L006** | **24h_post_challenge_FSW+_OsHV-1_1** | **3,7E+07** | **67,1** | **8017** | **0,323** | **1,121** | **0,050** |
| **115_S21_L006** | **24h_post_challenge_FSW+_OsHV-1_2** | **3,8E+07** | **67,5** | **8973** | **0,352** | **0,819** | **0,064** |
| **116_S22_L006** | **24h_post_challenge_FSW+_OsHV-1_3** | **4,9E+07** | **66,9** | **10803** | **0,330** | **1,024** | **0,055** |
| 118_S23_L006 | 24h_post_challenge_FSW+_control_1 | 3,8E+07 | 68,2 | 7927 | 0,305 | 0,002 |  |
| 119_S24_L007 | 24h_post_challenge_FSW+_control_2 | 3,8E+07 | 68,4 | 8081 | 0,309 | 0,001 |  |
| 120_S25_L007 | 24h_post_challenge_FSW+_control_3 | 3,2E+07 | 67,9 | 7288 | 0,336 | 0,001 |  |
| **EXP2** |  |  |  |  |  |  |  |
| SRR6679090 | AF11-T0-replicate | 5,1E+07 | 57,57 | 4646 | 0,157 | 0,000 |  |
| SRR6679091 | AF11-T0-replicate | 5E+07 | 59,99 | 4818 | 0,161 | 0,000 |  |
| SRR6679093 | AF11-T0-replicate | 5,1E+07 | 59,26 | 4823 | 0,159 | 0,000 |  |
| SRR6679086 | AF11-T6-replicate | 4,7E+07 | 58,83 | 4308 | 0,155 | 0,000 |  |
| SRR6679088 | AF11-T6-replicate | 5,3E+07 | 59,43 | 5026 | 0,159 | 0,000 |  |
| SRR6679089 | AF11-T6-replicate | 4E+07 | 59,55 | 3972 | 0,165 | 0,000 |  |
| **SRR6679084** | **AF11-T12-replicate** | **4,3E+07** | **58,81** | **4371** | **0,173** | **0,221** | **0,002** |
| **SRR6679085** | **AF11-T12-replicate** | **6E+07** | **59,57** | **6380** | **0,179** | **0,161** | **0,005** |
| **SRR6679087** | **AF11-T12-replicate** | **5E+07** | **58,46** | **5227** | **0,179** | **0,518** | **0,005** |
| **SRR6679058** | **AF11-T24-replicate** | **5,4E+07** | **56,76** | **5707** | **0,185** | **2,041** | **0,018** |
| **SRR6679059** | **AF11-T24-replicate** | **4,9E+07** | **57,83** | **4972** | **0,177** | **1,206** | **0,032** |
| **SRR6679061** | **AF11-T24-replicate** | **4,1E+07** | **57,27** | **4331** | **0,186** | **1,411** | **0,027** |
| SRR6679054 | AF11-T48-replicate | 6,3E+07 | 59,80 | 6188 | 0,166 | 0,000 |  |
| SRR6679055 | AF11-T48-replicate | 5E+07 | 59,42 | 4744 | 0,160 | 0,000 |  |
| SRR6679060 | AF11-T48-replicate | 4,8E+07 | 59,65 | 4598 | 0,162 | 0,000 |  |
| **SRR6679053** | **AF11-T60-replicate** | **4,9E+07** | **58,12** | **5516** | **0,192** | **1,062** | **0,032** |
| **SRR6679056** | **AF11-T60-replicate** | **5,6E+07** | **57,02** | **6574** | **0,207** | **1,493** | **0,030** |
| **SRR6679057** | **AF11-T60-replicate** | **4,8E+07** | **56,67** | **5387** | **0,198** | **1,598** | **0,033** |
| SRR6679052 | AF11-T72-replicate | 4,5E+07 | 59,73 | 4616 | 0,171 | 0,000 |  |
| SRR6679082 | AF11-T72-replicate | 4,3E+07 | 59,93 | 4669 | 0,181 | 0,000 |  |
| SRR6679083 | AF11-T72-replicate | 4,4E+07 | 59,38 | 4360 | 0,167 | 0,000 |  |
| SRR6679076 | AF21-T0-replicate | 5,2E+07 | 59,01 | 4750 | 0,155 | 0,000 |  |
| SRR6679077 | AF21-T0-replicate | 5,3E+07 | 59,67 | 4773 | 0,150 | 0,000 |  |
| SRR6679079 | AF21-T0-replicate | 6,1E+07 | 58,87 | 6186 | 0,173 | 0,000 |  |
| SRR6679072 | AF21-T6-replicate | 5,3E+07 | 59,34 | 4961 | 0,159 | 0,000 |  |
| SRR6679073 | AF21-T6-replicate | 4,2E+07 | 59,75 | 3991 | 0,157 | 0,000 |  |
| SRR6679078 | AF21-T6-replicate | 4,5E+07 | 59,06 | 4228 | 0,158 | 0,000 |  |
| SRR6679074 | AF21-T12-replicate | 4,8E+07 | 59,53 | 4934 | 0,173 | 0,001 |  |
| SRR6679075 | AF21-T12-replicate | 5,1E+07 | 59,99 | 5379 | 0,175 | 0,000 |  |
| SRR6679081 | AF21-T12-replicate | 4,8E+07 | 60,14 | 5341 | 0,186 | 0,000 |  |
| SRR6679068 | AF21-T24-replicate | 5,7E+07 | 59,95 | 7036 | 0,207 | 0,018 |  |
| SRR6679069 | AF21-T24-replicate | 5E+07 | 59,74 | 5702 | 0,190 | 0,000 |  |
| SRR6679080 | AF21-T24-replicate | 4,7E+07 | 59,61 | 5145 | 0,182 | 0,000 |  |
| SRR6679065 | AF21-T48-replicate | 5E+07 | 59,80 | 4804 | 0,161 | 0,000 |  |
| SRR6679066 | AF21-T48-replicate | 4,2E+07 | 59,25 | 4125 | 0,166 | 0,000 |  |
| SRR6679067 | AF21-T48-replicate | 5E+07 | 59,76 | 5147 | 0,171 | 0,000 |  |
| SRR6679062 | AF21-T60-replicate | 4,3E+07 | 59,98 | 4610 | 0,179 | 0,001 |  |
| SRR6679063 | AF21-T60-replicate | 5,9E+07 | 59,20 | 6120 | 0,175 | 0,000 |  |
| SRR6679064 | AF21-T60-replicate | 5,9E+07 | 59,45 | 6583 | 0,188 | 0,006 |  |
| SRR6679070 | AF21-T72-replicate | 5,6E+07 | 59,15 | 5492 | 0,166 | 0,000 |  |
| SRR6679071 | AF21-T72-replicate | 3,7E+07 | 59,71 | 3961 | 0,178 | 0,000 |  |
| SRR6679092 | AF21-T72-replicate | 5,6E+07 | 59,47 | 5947 | 0,178 | 0,000 |  |
