# Supplementary material for: ADAR-Editing during Ostreid Herpesvirus 1 Infection in Crassostrea gigas: Facts and Limitations
Source: mSphere. 2022 Apr 5;7(2):e00011-22. doi: 10.1128/msphere.00011-22 (PMC9044936; doi:10.1128/msphere.00011-22)

**Supplementary Figure 2.** ADAR and hyper-editing in EXP2. The expression levels of oyster ADAR1 are (dotted line, secondary axis, Transcripts Per Million) together with the level of hyper-editing (histogram, primary axis, ‰ of hyper-edited over mapped reads) are reported for EXP2 samples, averaged per experimental condition (N=3). Black refers to susceptible (S) oyster samples and grey to resistant (R) ones.


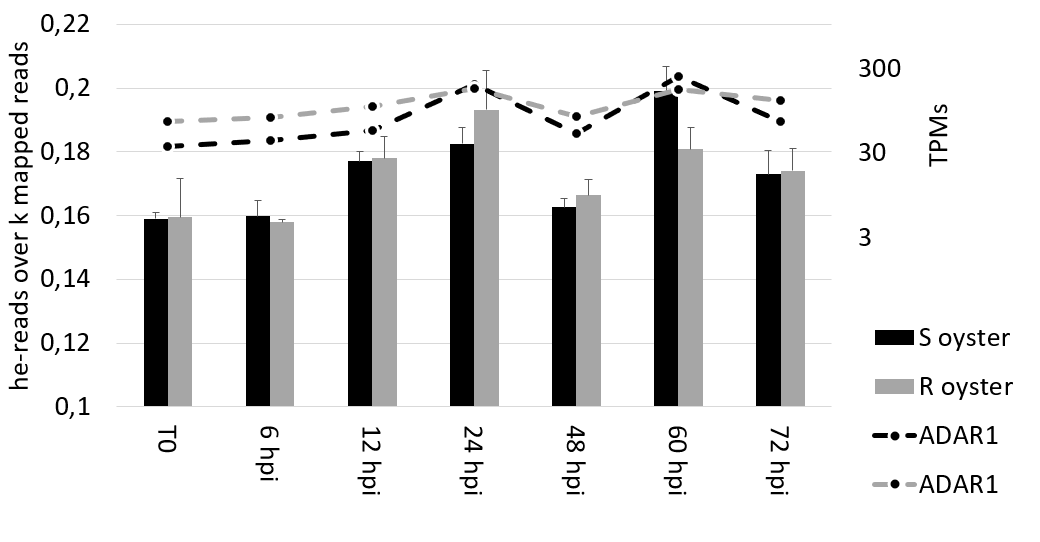

Supplement: FIG S2 [file msphere.00011-22-sf002.docx]
