# Supplementary material for: ADAR-Editing during Ostreid Herpesvirus 1 Infection in Crassostrea gigas: Facts and Limitations
Source: mSphere. 2022 Apr 5;7(2):e00011-22. doi: 10.1128/msphere.00011-22 (PMC9044936; doi:10.1128/msphere.00011-22)

**Supplementary Figure 3.** The distribution of the nucleotide variations in hyper-edited reads, for EXP1 (a) and EXP2 (b) samples.


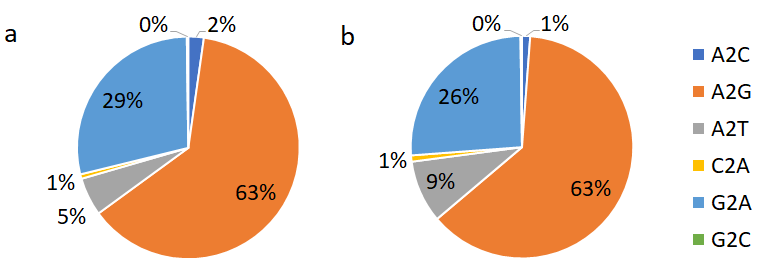

Supplement: FIG S3 [file msphere.00011-22-sf003.docx]
